# Dissociable contributions of ventromedial prefrontal and posterior parietal cortex to value-guided choice

**DOI:** 10.1016/j.neuroimage.2014.06.005

**Published:** 2014-10-15

**Authors:** Gerhard Jocham, P. Michael Furlong, Inga L. Kröger, Martin C. Kahn, Laurence T. Hunt, Tim E.J. Behrens

**Affiliations:** a FMRIB Centre, University of Oxford, John Radcliffe Hospital, Oxford OX3 9DU, United Kingdom; b Department of Experimental Psychology, University of Oxford, South Parks Road, Oxford OX1 3UD, United Kingdom; c Wellcome Trust Centre for Neuroimaging, University College London, 12 Queen Square, London WC1N 3BG, United Kingdom; d The Robotics Institute, Carnegie Mellon University, Pittsburgh, PA, USA; e Department of Systems Neuroscience, University of Hamburg, UKE, Martinistrasse 52, 20246 Hamburg, Germany; f Graduate Programme in Neuroscience, University of Oxford, United Kingdom; g Center for Behavioral Brain Sciences, Otto-von-Guericke-Universität Magdeburg, Germany

**Keywords:** Decision making, fMRI, Parietal cortex, Reward, Ventromedial prefrontal cortex

## Abstract

Two long-standing traditions have highlighted cortical decision mechanisms in the parietal and prefrontal cortices of primates, but it has not been clear how these processes differ, or when each cortical region may influence behaviour. Recent data from ventromedial prefrontal cortex (vmPFC) and posterior parietal cortex (PPC) have suggested one possible axis on which the two decision processes might be delineated. Fast decisions may be resolved primarily by parietal mechanisms, whereas decisions made without time pressure may rely on prefrontal mechanisms. Here, we report direct evidence for such dissociation. During decisions under time pressure, a value comparison process was evident in PPC, but not in vmPFC. Value-related activity was still found in vmPFC under time pressure. However, vmPFC represented overall input value rather than compared output value. In contrast, when decisions were made without time pressure, vmPFC transitioned to encode a value comparison while value-related parameters were entirely absent from PPC. Furthermore, under time pressure, decision performance was primarily governed by PPC, while it was dominated by vmPFC at longer decision times. These data demonstrate that parallel cortical mechanisms may resolve the same choices in differing circumstances, and offer an explanation of the diverse neural signals reported in vmPFC and PPC during value-guided choice.

## Highlights

• Value parameter represented in ventromedial PFC depends on available decision time.
• Under time pressure, vmPFC activity represents overall input value.
• Without time pressure, vmPFC transitions to encode a value comparison.

## Introduction

The ability to decide on appropriate courses of action amongst competing alternatives is central to adaptive success. Whilst neural signals representing the potential value of different courses of action are widespread throughout the brain (Cai et al., 2011, Dorris and Glimcher, 2004, Hernandez et al., 2002, Kable and Glimcher, 2007, Kim et al., 2008, Padoa-Schioppa and Assad, 2006, Platt and Glimcher, 1999, Serences, 2008, Sugrue et al., 2004, Wunderlich et al., 2009), two cortical regions, the ventromedial prefrontal cortex (vmPFC) and the posterior parietal cortex (PPC), have attracted particular attention for their likely roles in the selection process. Evidence for central roles in choice for these two brain regions comes from two independent and largely separate traditions. Extensive single unit recordings in the lateral intraparietal sulcus (LIP) of macaque monkeys during saccadic decisions have revealed activity that integrates sensory information to solve ambiguous sensory decisions (Gold and Shadlen, 2007); that tracks the relative value of competing actions (Platt and Glimcher, 1999, Sugrue et al., 2004) and the Bayesian evidence for different value-guided choices (Yang and Shadlen, 2007). By contrast, vmPFC's importance for value-guided choice has been established largely in the human literature. Patients with lesions to vmPFC become indecisive about even trivial decisions (Barrash et al., 2000); choices that *are* made are often made poorly (Bechara et al., 1994, Bechara et al., 2000) and according to unusual strategies (Fellows, 2006). In human imaging experiments, neural activity in this region often contains value representations consistent with a decision (Basten et al., 2010, Boorman et al., 2009, Jocham et al., 2012, Kolling et al., 2012); and the balance of excitatory and inhibitory neurotransmitters in vmPFC impacts both on this neural signature and on behaviour in a fashion consistent with competitive models of choice (Jocham et al., 2012).

These findings suggest analogous roles in choice for PPC and vmPFC. Such similarities are further strengthened by the finding that vmPFC lesions also impair decision-making in macaques (Noonan et al., 2010); and that, in humans, the same signatures of categorical choice can be seen in magnetoencephalography (MEG) signals from these two cortical regions (Hunt et al., 2012). The existence of two such similar neural signatures in two brain regions so distinct in both their anatomical location and connectivity pattern (Öngür and Price, 2000, Sack, 2009) raises the question of what distinguishes neural processing in vmPFC and PPC, and in what situations either region might come to the fore to influence decision-making. One intriguing possibility comes from the aforementioned MEG study. Here vmPFC involvement was strongest in trials early in the experiment, and stronger in trials that required integration across choice dimensions. In both cases, more vmPFC activity was associated with longer reaction times, possibly as a result of more deliberate and less automated choices. These data provided a suggestive hint that vmPFC and PPC might be capable of performing the same computations, but do so under differing circumstances. We therefore designed an experiment to explicitly test the hypothesis that vmPFC and PPC would perform decision-related computations in choice situations without or with time pressure, respectively.

## Methods

### Participants

31 healthy participants (11 females, aged 18 to 35 years) participated in the experiment. Written informed consent was obtained prior to the study. All experimental procedures were approved by the Central University Research Ethics Committee. Volunteers were paid between £ 20 and £ 30, depending on task performance. Three volunteers had to be excluded because of extreme head motion, leaving a final sample of 28 subjects (10 females).

### Behavioural task

**Fig. 1:**
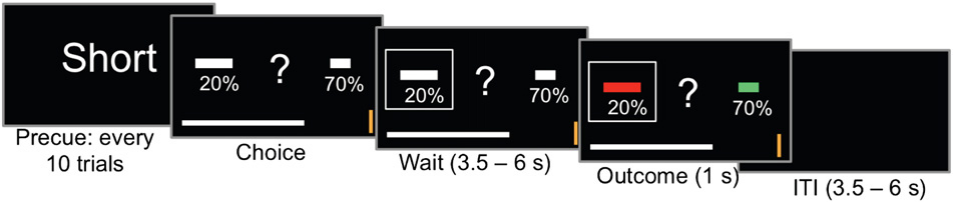
Task schematic. Short, middle and long trials were grouped in alternating blocks of 10 trials. Every 10 trials, a precue signalled the condition for the next 10 trials. In the short and middle condition, subjects could respond as soon as the options were onscreen. In the long condition, there was a fixed viewing period of 3 s before the central question mark appeared, prompting them to respond within 1 s.

During fMRI, subjects performed a task that involved repeatedly choosing between a left and right option to obtain monetary reward (Fig. 1). Each option consisted of one rectangular horizontal bar and a percentage written underneath it. The bar width represented the reward magnitude and the percentage specified the probability with which this reward would be delivered. Reward probabilities were independent, such that on any given trial, either one of the two options, both or none of them could be rewarded. The task thus required subjects to integrate reward probability and magnitude into a value estimate to make the best possible choice. Subjects made choices by pressing a left or right button with the index or middle finger, respectively, of the right hand. When a reward was available for the chosen option, an amount proportional to the reward magnitude was added to a gray progress bar at the bottom of the screen. Subjects' goal was to move the progress bar across a gold target line to the right to win £ 2, at which time the progress war was reset to zero and subjects started over again. On a subset of trials, which we refer to as ‘no brainer’ trials, both the magnitude and probability of one option were higher than on the alternative option. The reward schedule was designed such that the correlation between chosen and unchosen value was as low as possible, thus allowing for largely separate portions of variance to be explained by those factors. The mean correlation of these two factors was *r* = 0.18.

In the *short* condition, options were presented on screen and subjects had to make a choice within one second. In the *middle* condition, options were presented on screen and subject could make a decision whenever they wanted, without any response deadline. In the *long* condition, options were first presented for a fixed viewing period of 3 s before a question mark appeared, from which time subjects had 1 s to respond. If subjects failed to respond within the 1 s response window in the *short* and *long* conditions, the following message appeared on the screen: “Please respond faster!”. After a response was made, the selected option was highlighted by a grey frame around the chosen option, which remained on screen for 3.5 to 6 s until the outcome was revealed for 1 s. The outcome (reward or non-reward) was indicated by the bars turning green or red, respectively. On every trial, the outcomes of both options were revealed. The outcome was followed by an intertrial interval (blank screen) of 3.5 to 6 s. Short, middle and long trials were administered in alternating blocks of 10 trials. After 10 trials of one condition were completed, a precue with the message short, middle or long appeared on screen for 1 s. 70 trials of each condition were completed. Thus, trials in the three conditions were identical, except for the timing manipulation, which lead to different decision times (median decision time = 793, 1180 and 3366 ms, for the *short*, *middle* and *long* conditions, respectively).

In each condition, we searched for two possible neural signals, which are argued to represent different aspects of valuation and choice. An fMRI signal that correlates with the sum of available values is argued to represent a stimulus valuation stage that comes before a decision process (Hare et al., 2009, Hare et al., 2011a, Hare et al., 2011b, Hunt et al., 2012, Plassmann et al., 2007, Plassmann et al., 2010). By contrast, an fMRI signal that correlates with the difference between chosen and unchosen values is argued to reflect the outcome of the decision process itself (Basten et al., 2010, Boorman et al., 2009, FitzGerald et al., 2009, Hunt et al., 2012, Jocham et al., 2012, Kolling et al., 2012), as it requires the computation of which option has been chosen and which option remains unchosen. Indeed, if decision-related activity is imaged at millisecond resolution, a clear transition from value sum to value difference correlations can be seen as the decision unfolds (Cai et al., 2011, Hunt et al., 2012). Network models of decision making imply that this transition occurs because over time, the representation of the unchosen option changes. Thus, while initially, network activity correlates positively with the value of both options, it is the unchosen option which becomes suppressed, thereby resulting in a positive correlation between unchosen value and network activity. Our tests therefore focus on the effects of unchosen value.

Here, by simply manipulating the amount of time that subjects spend making decisions, we are able to change the types of value coding that can be seen in vmPFC and PPC. We find that under time pressure vmPFC encoded value sum, whereas PPC encoded value difference. Without time pressure, vmPFC encoded value difference, whereas coding of value-related parameters completely disappeared from PPC. Under time pressure, behaviour was guided primarily by PPC, whereas it came under control of vmPFC without time pressure.

### MRI data acquisition

MRI data were acquired on a 3 T Siemens Verio system equipped with a 32 channel phased array head coil (Siemens, Germany). First, a high-resolution T1-weighted scan was acquired using an MPRAGE sequence. For functional imaging, 45 slices with a voxel resolution of 3 mm isotropic were obtained (no gap) using a sequence optimized for the orbitofrontal cortex (Deichmann et al., 2003), with TR = 3000 ms, TE = 30 ms, flip angle = 87°, a slice angle of 15° and a local *z*-shim applied around the region of the orbitofrontal cortex. Field maps were acquired using a dual echo 2D gradient echo sequence with TR = 488 ms and TE of 7.65 ms and 5.19 ms on a 64 × 64 × 40 grid. A total of 881 volumes were acquired on average, depending on subjects' reaction times, thus resulting in a total task duration of about 44 min. We used Presentation (Neurobehavioural Systems, USA) to present the task and record subjects' behaviour.

### Behavioural data analysis

Prospect theory (Tversky and Kahneman, 1992) has demonstrated that humans do not weight magnitudes and probabilities in a statistically optimal way. We derived the subjective reward magnitudes and probabilities by fitting utility functions according to prospect theory:

$$\begin{array}{l} r_{S}\;=\;{r_{O}}^{\alpha }\\ p_{S}\;=\;{p_{O}}^{\gamma }/{\left({p_{O}}^{\gamma }\;+\;{\left(1\;–\;p_{O}\right)}^{\gamma }\right)}^{1/\gamma } \end{array}$$

where *r*_O_ and *p*_O_ are the objective reward magnitude and probability that are transformed into the subjective magnitude and probability, *r*_S_ and *p*_S_, respectively. These values can then be used to calculate subjective expected values as

$$\mathrm{sEV}\;=\;r_{S}*p_{S}$$

The modelled probability to choose either of the two options was then given by a softmax choice rule:

$$P_{\left(C=K\right)}\;=\;\exp\left(\mathrm{sE}V_{K}*\tau \right)/\;\left(\exp\left(\mathrm{sE}V_{L}*\tau \right)\;+\;\exp\left(\mathrm{sE}V_{R}*\tau \right)\;\right)$$

where *K* is the choice made by the subject (left or right), *n* is the number of options available, and *τ* is the softmax temperature that expresses the degree of stochasticity in subjects' behaviour. We used a Bayesian estimation procedure custom-implemented in MATLAB (The Mathworks, USA) to obtain the free parameters *α*, γ and τ that best describe subjects' behaviour. The parameter space was set up as a three-dimensional grid in log space with 150 points in each dimension. The joint posterior distribution of the unknown model parameters is then given by the product of choice probabilities over trials under each possible parameter combination in the grid. The marginal posterior distributions on each parameter were then obtained by marginalizing (numerical integration) over the remaining two dimensions of the grid. The optimal parameters were then taken as the distribution means of those marginal posterior distributions. For the analysis of fMRI data, we used sEV, rather than the objective pascalian values, as the former have been found to provide a better fit to neural data in reward-guided choice tasks (Hsu et al., 2009).

### MRI data analysis

Analysis of fMRI data was performed using tools from the Functional Magnetic Resonance Imaging of the Brain (FMRIB) Software Library (FSL (Smith et al., 2004)). Functional data were motion-corrected using rigid-body registration to the central volume (Jenkinson et al., 2002), corrected for geometric distortions using the field maps and an n-dimensional phase-unwrapping algorithm (Jenkinson, 2003), high-pass filtered using a Gaussian-weighted lines 1/100 Hz filter and spatial smoothing was applied using a Gaussian filter with 6 mm full-width at half maximum. Conservative independent component analysis was carried out using MELODIC (Damoiseaux et al., 2006) to identify and remove obvious artefacts. EPI images were registered with the high-resolution brain images and normalized into standard (MNI) space using affine registration (Jenkinson and Smith, 2001). A general linear model was fitted into prewhitened data space to account for local autocorrelations (Woolrich et al., 2001). To investigate activity related to the value difference between the chosen and unchosen options, we set up a single GLM that contained the following seven regressors: value difference, value sum, outcome value (reward vs. no reward obtained), one regressor for the main effect from stimulus presentation to response, one regressor for the main effect of outcome phase, and two stick functions modelling left and right button presses, respectively. In addition, the six motion parameters from the motion correction were included in the model. A second GLM was set up to decompose the effects of value sum and value difference into their constituent terms, chosen value and unchosen value. To this end, the second GLM was identical to the first one, except that the regressors for value sum and value difference were replaced by regressors coding for the chosen and unchosen values. Contrast images from the first level were then taken to the group level using a random effects analysis. Results are reported at a threshold of *p* < 0.001, uncorrected.

Our analyses focused on two regions of interest (ROI), the vmPFC and a region in the PPC, the posterior superior parietal lobule (pSPL), as those had been shown to be related to key decision variables in a previous study using MEG (Hunt et al., 2012). In order to obtain independent regions of interest (ROIs) that were not subject to selection bias, we selected ROIs on the basis of a previous study using an experimental paradigm that was identical to the current study (Jocham et al., 2012), except for the variations in the allowed decision time. From this study, we thresholded the contrast for value difference at *p* < 0.001 and used the resulting activation in the vmPFC as ROI. To obtain an ROI for the pSPL, we used the same contrast, but set the inclusion threshold to be more liberal at *p* < 0.01. This resulted in ROI sizes of 648 mm^3^ (vmPFC) and 1288 mm^3^ (pSPL). We think that the sub-threshold activation of pSPL observed in this and other previous studies from our lab is exactly a consequence of pSPL only becoming recruited when choices are made very quickly. Note that the above-described whole-brain analyses serve a merely descriptive purpose to highlight that we can detect activations in our present study that overlap with our independently defined ROIs. In agreement with previous studies (Boorman et al., 2009, Boorman et al., 2011, Hunt et al., 2012), we find a value difference correlate in the vmPFC (MNI *x* = − 2, *y* = 28, *z* = − 18, *z*-max = 4.03) and bilaterally in the pSPL (MNI *x* = 16, *y* = − 48, *z* = 56, *z*-max = 3.15 and *x* = − 12, *y* = − 52, *z* = 60, *z*-max = 3.14, Fig. 4). Note the very close correspondence of these activations with both our independent ROI and with the coordinates of other studies reporting an overall value signal (Hare et al., 2009, Plassmann et al., 2007, Plassmann et al., 2010) and value difference signal (FitzGerald et al., 2009, Kolling et al., 2012) in vmPFC. In addition to these a priori ROI, we also find activation in the posterior cingulate cortex (PCC, MNI *x* = − 6, *y* = − 46, *z* = 34, *z*-max = 3.72). We report the data from this PCC ROI in the supplementary material, along with a fourth region, the midcingulate cortex (MCC), which was found in the previous, but not in the current study.

### ROI analyses

We extracted raw BOLD signal timecourses from the above ROI. The timeseries of each volunteer was then cut into trials with a duration of 16 s, where options were presented at 0 s, the response was made at 0.77, 1.41 or 3.4 s (for short, middle and long) and the outcome was presented at 5.56, 6.21 or 8.24 s (for short, middle and long), which corresponds to the mean onsets of these events across subjects and trials. Timeseries were resampled to a resolution of 300 ms using cubic spline interpolation in MATLAB. A GLM containing the parameters of interest was then fitted at each time point for each volunteer. The two GLM used here are the same as described above for the whole-brain analysis. In addition, reaction time was added as a covariate of no interest. This resulted in a timecourse of effect size for each regressor in the design matrix and for each volunteer. These timecourses were then averaged across participants. For statistical testing, a hemodynamic response function was then fit to these effect size timecourses from each participant (Behrens et al., 2008, Boorman et al., 2011). This resulted in one parameter estimate per effect size timecourse and participant. These parameter estimates were tested for statistical differences from zero and between conditions.

## Results

### Behaviour

**Fig. 2:**
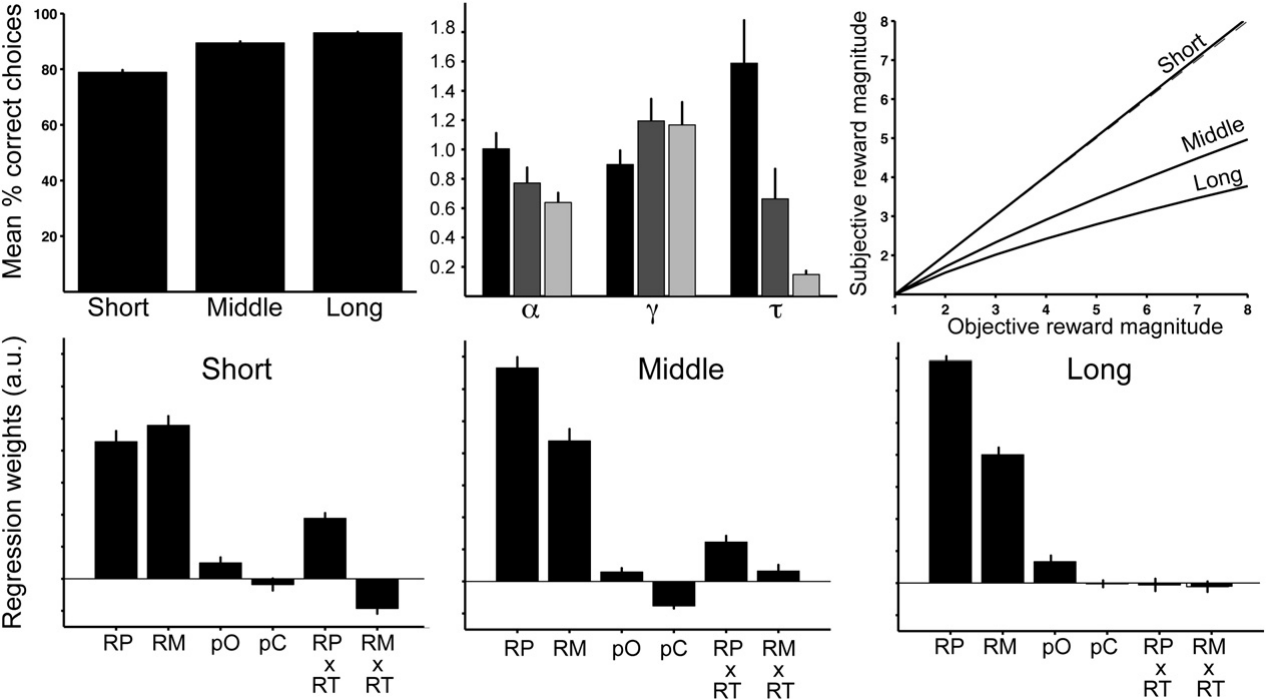
Behavioural results for the three conditions. A) Percent choices of the higher value option. B) Parameters from prospect theory for subjective weighting of reward magnitude (*α*) and probability (*γ*), and softmax temperature (*τ*). C) Plotting the objective versus subjective magnitudes using the weighting parameter displayed in (B) shows underweighting of reward magnitudes in the middle and long, but not short condition. D–F) Regression of experimental parameters against subjects' choice. Positive values indicate that the parameter increases the probability to select the option. RP: reward probability, RM: reward magnitude, pO: outcome on previous trial, pC: choice on previous trial, RP × RT: interaction between reward probability and reaction time, RM × RT: interaction between reward magnitude and reaction time.

As expected, reaction times were faster in *short* (median = 793 ms) compared to *middle* (median = 1180 ms, *t*_27_ = 8.3, *p* < 0.0001). In *long*, reaction times were by definition longer (median = 3366 ms) due to the imposed 3 s waiting period. We found that choice accuracy depended on the time allowed for a choice (Fig. 2A). The percentage of choices of option with the higher objective value was significantly higher in *long* compared to *short* and *middle* (*t*_27_ > 4.54, *p* < 0.00015) and higher in middle compared to short (*t*_27_ = 4.04, *p* < 0.0004). We used prospect theory (Tversky and Kahneman, 1992) to characterize subjects' behaviour in this task. Whereas optimal behaviour on the task would be to multiply magnitude and probability and to choose the option with the highest Pascalian value, the model has two parameters that warp probability and reward space to match subject behaviour. A third parameter, the softmax temperature, *τ*, reflects the accuracy of subject decisions on difficult trials (trials with low value difference). *τ* was higher in *short* compared to *middle* and *long* (*t*_27_ > 2.6, *p* < 0.015), and higher in *middle* compared to *long* (*t*_27_ = 2.57, *p* = 0.016). This means that in *short*, subjects performed particularly poorly compared to *middle* and *long* on trials where the value difference between options was small. Notably, the parameter that describes subjective distortions of reward magnitude (*α*) also differed between conditions (Fig. 2B and C). In *short*, *α* did not differ from 1 (*p* > 0.96), indicating that subjects' subjectively distorted reward magnitudes were identical to the objective magnitudes. In contrast, there was pronounced under-weighting of reward magnitudes (*α* < 1) in *middle* (*t*_27_ = 2.06, *p* = 0.049) and *long* (*t*_27_ = 5.08, *p* < 0.0001). Between-condition comparison also showed that *α* was higher in *short* compared to *long* (*p* = 0.007) and, by trend compared to *middle* (*p* = 0.075). No such differences were found for the parameter that describes the distortion of reward probability (*γ*). To investigate this pattern in more detail, we performed a regression of different parameters that could influence participants' decisions against their choices. The above results suggest that subjects' decisions are guided to an equal extent by probability and magnitude in *short*, whereas in *middle* and *long*, they appear to base their choices primarily on reward probability while tending to neglect reward magnitudes. If this is the case, the regression coefficients for probability should be equal to those for magnitudes in *short*, but higher in *middle* and *long*. Fig. 2D–F shows exactly such a pattern. The regression coefficients for probability were significantly larger than those for magnitude in *middle* (*t*_27_ = 3.39, *p* = 0.002) and *long* (*t*_27_ = 7.76, *p* < 0.0001), while they did not differ in *short* (*p* > 0.4). The differences between the probability and magnitude regression coefficients also differed between *short* and *middle* (*t*_27_ = 5.39, *p* < 0.0001) and *short* and *long* (*t*_27_ = 6.67, *p* < 0.0001). Further to these differences between conditions, we also found that within both *short* and *middle*, there was a positive interaction effect of probability and reaction time on choice (*t*_27_ = 5.6, *p* < 0.0001). This means that even in *short*, with increasing decision times, subjects put more weight on reward probabilities. As expected, no such interaction with reaction time was found in *long*, due to the imposed pre-response waiting period.

**Fig. 3:**
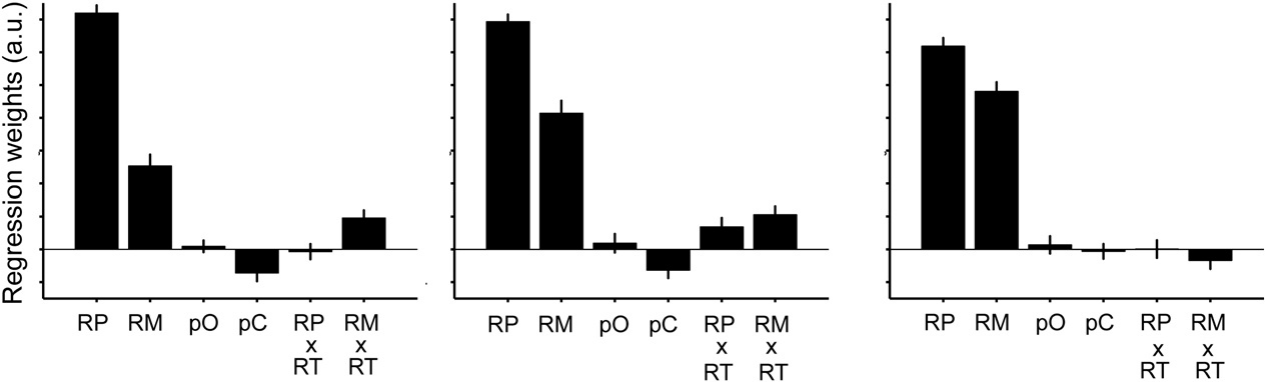
Results for the behavioural control experiment. Bars represent regression coefficients (mean ± SEM) obtained from a regression of experimental parameters against subjects' choices. Positive values indicate that the parameter increases the probability to select the option. RP: reward probability, RM: reward magnitude, pO: outcome on previous trial, pC: choice on previous trial, RP × RT: interaction between reward probability and reaction time, RM × RT: interaction between reward magnitude and reaction time.

However, we questioned whether this behavioural pattern could be due to the specific way the task was presented. In our experiment, reward magnitudes were displayed as rectangular bars, whereas probabilities were displayed as percentage numbers. It is possible that the bars can be processed faster than the numbers at a perceptual level. This would imply that the behavioural effect is simply due to the fact that subjects did not have enough time to process probabilities in *short*. We performed a behavioural control experiment in which the option display was reversed, such that probabilities were now shown as bars and magnitudes were shown as numbers. Under these conditions, we find that in *short*, subjects' choices are governed by both reward probability and magnitude, but to a much larger extent by probability (*t*_27_ = 8.18, *p* < 0.0001). This dominance of probability, while still present in *middle* and *long*, was reduced compared to *short* (comparison of the difference between the probability and magnitude weights: *t*_27_ = 3.62 *p* < 0.003 for *short* versus *middle* and *long*, and *middle* versus *long*, Fig. 3). Furthermore, the reward magnitude distortion parameter *α* now also showed the reverse pattern: *α* was lower than 1 in *short* and *middle* (*t*_27_ = 4.1 *p* < 0.0015, but not in *long* (*p* > 0.67). This additional data suggests that the differential weighting of decision variables across conditions is primarily driven by choices being governed by the options' perceptual features (bar width) under time pressure.

### Cortical value correlates

**Fig. 4:**
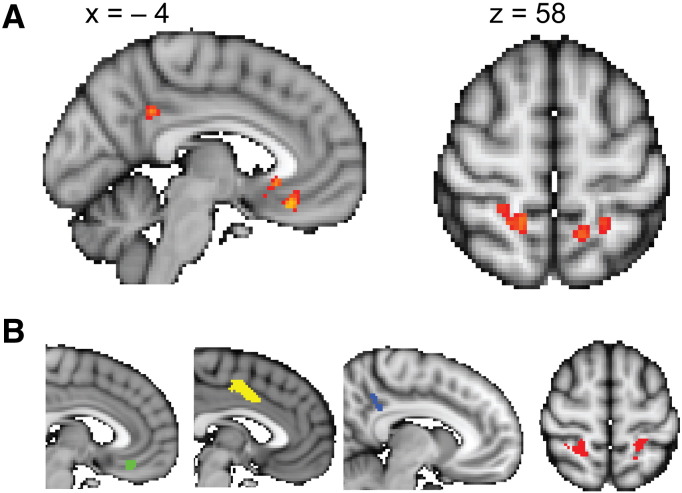
Whole-brain results and masks used for ROI analyses. A) Effect of value difference at *p* < 0.001 in the vmPFC and posterior cingulate (left) and, at lower threshold (*p* < 0.01) in the pSPL (right). B) Regions of interest selected from a previous study showing activity related to value difference in the vmPFC (green), midcingulate cortex (yellow), posterior cingulate cortex (blue) and pSPL (red).

Our analyses focused on two regions of interest (ROI), the vmPFC, a region in the PPC, the posterior superior parietal lobule (pSPL) as those had been shown to be related to key decision variables in a previous study using MEG (Hunt et al., 2012). We report two additional areas in the supplementary materials, the midcingulate cortex (MCC) and the posterior cingulate cortex (PCC), which showed value-related activity in a previous study but about which we had no a priori hypothesis. We first performed a whole-brain analysis investigating the effects of value difference across conditions (see Methods). We found a value difference correlate in the vmPFC (MNI *x* = − 2, *y* = 28, *z* = − 18, *z*-max = 4.03), in posterior cingulate cortex (PCC, MNI *x* = − 6, *y* = − 46, *z* = 34, *z*-max = 3.72) and bilaterally in the pSPL (MNI *x* = 16, *y* = − 48, *z* = 56, *z*-max = 3.15 and *x* = − 12, *y* = –52, *z* = 60, *z*-max = 3.14, Fig. 4). Note that this whole-brain analysis primarily serves display purposes. The relevant statistical tests are performed directly on the ROI data.

Next, we extracted raw BOLD signal timecourses from the above ROI, divided it up into trial epochs and regressed design matrices containing the value-related parameters of interest against the BOLD signal at each timepoint in each trial (see Methods and materials), separately for the *short*, *middle* and *long* conditions. A first analysis tested for the presence of representations of either value sum or value difference in the two ROI in the different conditions by running separate *t*-tests on value sum and value difference. Next, to directly test our assumption that vmPFC and pSPL were differentially engaged in coding value-related parameters across conditions, we performed a two-way ANOVA with the within-subjects factors brain area (vmPFC, pSPL) and condition (short, middle, long) for the chosen and unchosen value effects. For the unchosen value, we found an effect of brain area (F_1,27_ = 5.72, *p* = 0.024) and a brain area × condition interaction (F_2,54_ = 3.94, *p* = 0.025), but no effect of condition alone (*p* = 0.30). We followed this up by post-hoc tests. We expected that in *short*, vmPFC activity would be positively correlated with both the value of the chosen and unchosen options, thus giving rise to a correlation with value sum. In contrast, in *middle* and *long*, we expected a positive correlation with chosen value, but a negative correlation with the unchosen value, thus giving rise to a correlation with value difference. Note that the crucial statistical test is on the effect of the unchosen value, as the key prediction of the biophysical model is that the unchosen value will be changed over time to correlate positively with vmPFC activity initially but then change to correlate negatively as the competition is resolved. In contrast, the correlation with the chosen value is not expected to change significantly over time. Hence, because value sum and value difference reflect both chosen and unchosen value effects, testing on the unchosen value is a more sensitive test for our hypothesis.

**Fig. 5:**
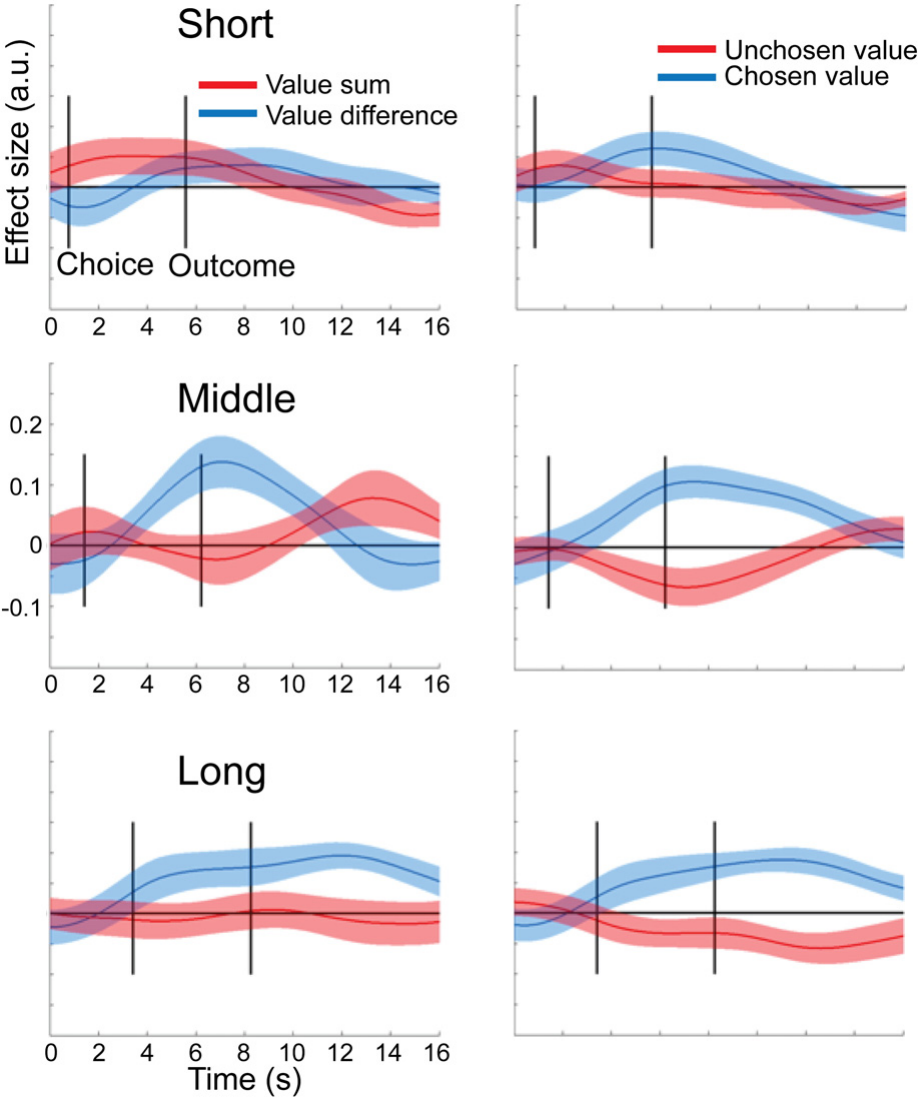
Timecourse of value-related effects in the ventromedial prefrontal cortex (vmPFC) over the course of a trial in the three conditions. Left column: effects of value difference and value sum on BOLD activity in vmPFC. Right column: Effects of chosen and unchosen option value on vmPFC BOLD activity. Solid lines represent mean effect sizes across participants, and shaded areas are standard error of the mean.

In vmPFC, we found exactly such a pattern. In *short*, there was an effect of value sum (*t*_27_ = 1.91, *p* = 0.033), but not of value difference (*p* > 0.1). In contrast, in *middle* and *long*, there wasn't any effect of value sum (*p* > 0.38) but instead vmPFC now encoded value difference (*t*_27_ = 2.19, *p* < 0.02, Fig. 5). According to our hypothesis, this should be due to the different effects of the unchosen value: While we expected a positive correlation with the chosen value in all three conditions, the unchosen value was hypothesized to be positively correlated with vmPFC activity in *short*, but negatively in *middle* and *long*. Direct pre-planned comparisons between conditions showed that the effect unchosen value was indeed more positive in short compared to middle (*t*_27_ = 2.05, *p* = 0.025) and, by trend, *long* (*t*_27_ = 1.54, *p* = 0.068).

**Fig. 6:**
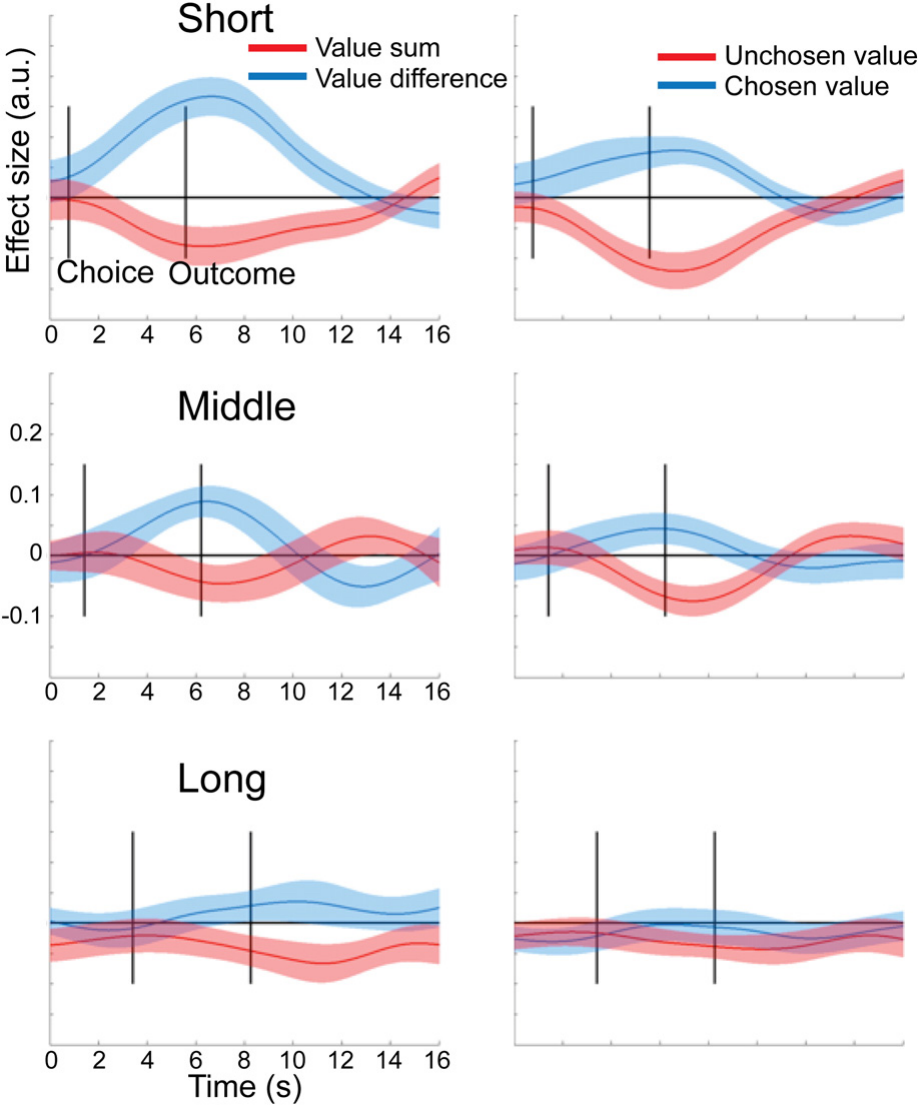
Timecourse of value-related effects in the posterior superior parietal lobule (pSPL) over the course of a trial in the three conditions. Left column: effects of value difference and value sum on BOLD activity in pSPL. Right column: Effects of chosen and unchosen option values on pSPL BOLD activity. Solid lines represent mean effect sizes across participants, and shaded areas are standard error of the mean.

This pattern of results contrasted with the pSPL. There was robust coding of value difference in the pSPL in *short* (*t*_27_ = 5.0, *p* < 0.0001) and *middle* (*t*_27_ = 2.34, *p* < 0.015), but this was absent in *long* (*p* > 0.27, Fig. 6). Focussing again on the unchosen value, we found that a negative effect was present in both *short* and *middle* (*t*_27_ > 3.25, *p* < 0.0015), but not in *long* (*p* > 0.29, Fig. 6). Again, direct pre-planned comparisons revealed a stronger negative unchosen value effect in *short* compared to *long* (*t*_27_ = − 2.72, *p* = 0.011), and a stronger negative effect in *short* compared to *middle* (*t*_27_ = 2.05, *p* = 0.025). Thus, pSPL showed a pattern opposite to that of vmPFC, with representation of value difference being pronounced under time pressure, but gradually diminishing as subjects were allowed more time to decide.

### Relationship between cortical value coding and performance

The above data suggest that at short decision times, choices are governed by pSPL whereas they come under control of the vmPFC at longer decision times. To test this, we set up (for each condition) a general linear model that contained the chosen and unchosen value effects from vmPFC and pSPL as regressors to predict choice performance (% choices of the higher-value option). In each of the three models, we set up one contrast for the effect of chosen value in both regions and one contrast directly comparing the effect of the chosen value between vmPFC and pSPL. In the *short* condition, decision accuracy tended to correlate positively with the pSPL chosen value signal (*t*_27_ = 1.32, *p* = 0.099), but not with the vmPFC chosen value signal (*t*_27_ = − 0.99, *p* > 0.16). Likewise, direct comparison between pSPL and vmPFC further showed that behaviour was, by trend, related more to the chosen value signal in pSPL than in vmPFC (*t*_27_ = 1.471, *p* = 0.076). This pattern reversed in the *middle* condition. Here, decision accuracy tended to correlate positively with the chosen value signal in vmPFC (*t*_27_ = 1.532, *p* = 0.069) but not in pSPL (*t*_27_ = − 0.69, *p* > 0.75). Direct comparison between pSPL and vmPFC showed that, in contrast to the *short* condition, behaviour was now more related to the chosen value signal in vmPFC than in pSPL (*t*_27_ = 1.74 *p* = 0.047). A similar pattern as in *middle* emerged in the *long* condition. Decision accuracy was positively related to the vmPFC (*t*_27_ = 1.94 *p* = 0.031), but not to the pSPL chosen value signal (*t*_27_ = − 0.195, *p* > 0.4). Again, direct comparison between vmPFC and pSPL showed, by trend, a stronger relationship of decision accuracy with vmPFC than with pSPL (*t*_27_ = 1.33 *p* = 0.097). Thus, overall it appears that value-guided choice is under stronger control by parietal rather than prefrontal cortex when subjects have to make very fast decisions, whereas prefrontal cortex seems to take a more important role when subjects do not face time pressure.

## Discussion

A number of neuroimaging studies have found correlates of value in the vmPFC. Yet, there has been disagreement about the computation supported by this region. Some of the studies found vmPFC BOLD to correlate with the value of the available options (Hare et al., 2009, Hare et al., 2011a, Hare et al., 2011b, Plassmann et al., 2007, Plassmann et al., 2010), whereas others found it to be correlated with either the value of the chosen option only (Wunderlich et al., 2009, Wunderlich et al., 2010), or with the value difference between chosen and unchosen options (Boorman et al., 2009, Jocham et al., 2012). This is a fundamental difference, as the former would reflect a signal that serves as an input to a decision process, which might then be implemented by some downstream brain structure. In contrast, a correlation with chosen value, or value difference, reflects a signature of a decision process itself, i.e. a categorical choice.

According to network models of decision making, neuronal pools in vmPFC initially reflect value-related inputs, thus giving rise to an overall value signal, i.e. a correlation with the value of both options. Neuronal competition then results in one pool ending up in a high-firing state (the neurons representing the option that is chosen), whereas activity in the other pool is suppressed (the neurons representing the unchosen option). Thus, depending on the time at which the network dynamics are observed, it is possible to find a correlate of either overall value or value difference within the same brain area. We have recently provided evidence for such a competition mechanism in the vmPFC. Neural dynamics in both vmPFC and pSPL recorded with MEG matched with these model predictions (Hunt et al., 2012). Furthermore, across-subject variations in the vmPFC levels of the major neurotransmitters GABA and glutamate predicted decision accuracy and the dynamics of the vmPFC value difference signal in a pattern as predicted by a network model (Jocham et al., 2012). However, this competition requires time to be resolved. If a choice is made very quickly, either because no integrative value comparison is required (as when deciding between a preferred and non-preferred food or on no-brainer trials) or because subjects are put under time pressure, the network still represents the overall value (Hunt et al., 2012). Our present results provide support for such a mechanism. When subjects are forced to respond very quickly, the competition within the vmPFC has not been resolved and hence it still represents value sum. In contrast, when more time for a decision was allowed, the competition has already been resolved and the network no longer represents overall value, but instead has made the transition to a correlation with value difference.

If the vmPFC represents value under time pressure, but does not transform these inputs into a choice, then the decision must be made by another brain region. A candidate region for this is a region in the PPC, the pSPL. We show that under time pressure, activity in pSPL correlates with value difference. As the value difference signal begins to emerge in vmPFC at longer decision times, correlates of value-related parameters entirely disappear from pSPL. This is in line with recent findings suggesting that vmPFC is not involved in choice in situations where decisions are made very quickly, on the basis of perceptual features. We recently showed that vmPFC coding of value difference disappeared over the course of an experiment as subjects' responses became incrementally faster. This was paralleled by increasing recruitment of the pSPL (Hunt et al., 2012). Likewise, a value difference correlate in vmPFC was not observed on so-called no-brainer trials, where both reward probability and magnitude dictated the same choice (Hunt et al., 2012). In other words, vmPFC was only involved when the trial required subjects to integrate probabilities and magnitudes into a combined value estimate. If no such integration is required, or if choices are highly overtrained, vmPFC appears not to be involved. Further to this, we recently showed that vmPFC levels of GABA and glutamate were predictive of subjects' choice performance. Importantly however, rather than correlating with overall choice accuracy, neurotransmitter levels were most predictive of subjects' propensity to select the higher-value option on difficult trials with low difference in the options’ values (Jocham et al., 2012). On the other hand, those fMRI studies finding a correlate of overall value rather than value difference either involve only a valuation process, as in willingness to pay studies (Plassmann et al., 2007, Plassmann et al., 2010, Sokol-Hessner et al., 2012), or again decisions that are likely very automated and hence can be made very quickly, such as when choices have to be made between food items (Hare et al., 2009, Hare et al., 2011a, Hare et al., 2011b), where subjects have clear preferences and hence need not rely on an effortful value comparison mechanism.

Our data thus offer a mechanistic explanation for the discrepant results across studies. They also suggest complementary roles for vmPFC and pSPL in decision-making. While pSPL affords the ability to respond quickly in the face of time pressure, or in situations that do not require much cognitive effort, vmPFC seems to be recruited when choices demand more time, possibly as a result of computing an integrated value. Apparently, and as suggested by biophysical models, this process is more time-consuming and hence does not dominate choice at very short response times. However, network models also suggest an alternative explanation. The models can be modified to allow faster decisions by increasing the degree of recurrent excitation. Under these conditions, the network still makes a decision, but activity primarily represents value sum rather than value difference (Hunt et al., 2012). This would suggest that vmPFC is involved in comparison in all three conditions, but that the signature of this comparison changes from *short* to *long*. Arguing against this interpretation, we find that choice accuracy was related more to value-related activity in pSPL than in vmPFC in the short condition, whereas this relationship reversed in the *middle* and *long* conditions. This varying correlation with behaviour, albeit not very pronounced, suggests that choices are indeed under differential control of the two brain regions under the differing experimental demands. However, despite the pronounced representation of chosen and unchosen value in the pSPL, there was only a rather weak relationship between value coding in pSPL and choice accuracy in the *short* condition. This hints at a likely involvement of additional brain regions in decision making under time pressure. It is also important to note that we do not know why the value representations change between pSPL and vmPFC across conditions. It is possible that time pressure enhances levels of stress or attention, and the resulting increase in release of acetylcholine and norepinephrine (or other stress-related neuromodulators or neurohormones) drives the change in neural representation. This will be a topic for future research in both theoretical and experimental studies.

It has to be noted that pSPL is located in a region of parietal cortex that has been involved in attentional mechanisms and in the control of hand and eye movements. Because eye movements and force of button presses were not measured in our study, it might be argued that our findings of value-related activity in pSPL are confounded by these factors. However, several points argue against such an interpretation. Firstly, we are not reporting differences in main effects between conditions and instead report only value correlates. Therefore, any differences that can simply be ascribed to different time pressure will not affect our results, only the interaction of time pressure with value will be affected. However, since we have included reaction times as covariates in all of our GLM analyses, the value correlates we report are orthogonal to reaction times. Secondly, attention and saccades are tightly linked to value comparison. There is evidence that the value comparison process is indeed guided by visual fixations (Krajbich et al., 2010) and explicitly manipulating participants' attention to one option made them more likely to select that option, independent of its value (Lim et al., 2011). Furthermore, the force of hand movements is directly related to an option's expected value (Pessiglione et al., 2007). Thus, attention, visual fixation and fervency of hand movements likely represent different facets of a value comparison process. Further to this, our pSPL ROI lies in an area slightly dorsal to what would correspond to primate LIP and is more likely equivalent to the monkey parietal reach region, and therefore likely more related to hand and arm rather than eye movements (Fearnley and Lees, 1991, Shiner et al., 2012). As stated above, the effects we report are orthogonal to reaction time, and hence likely to differences in movement speed.

We have reported differences between two brain regions involved in decision making. One might argue that such direct comparison between brain regions may be problematic because of potential differences in the hemodynamic response. However, it is crucial to note that we do not compare the absolute magnitude of the BOLD response. Instead, we compare the modulation of the BOLD signal by value, over and above the mean hemodynamic response. Furthermore, we show that vmPFC represents value difference when there is no time pressure, but not under time pressure, while the opposite pattern is found in pSPL. Such a double dissociation could not occur if one of the two brain regions simply had an overall lower neurovascular response. In such a case, this region would generally show reduced responding unter either condition.

It is notable that longer decision times were not only paralleled by an increase in decision accuracy, but also by differential weighting of reward magnitudes and probabilities. Our control experiment indicated that this was due to perceptual characteristics of the task. Apparently, under time pressure, decisions can be performed primarily by parietal cortex if the choices can be made on the basis of the options' perceptual features, either spatial or numerical (Hubbard et al., 2005, Pinel et al., 2004), such as the size of reward bar or the magnitude of the presented number. Only with more time, allowing for the calculation of internal subjective values, vmPFC comes to dominate the decision process.

In summary, we have demonstrated that the precise value correlate found in vmPFC depends on the network dynamics, which in turn are subject to the specifics of the experimental setup. Furthermore, our data point to complementary roles for vmPFC and pSPL in value-guided decision making. While pSPL enables rapid choices, vmPFC is important in more time-consuming decisions, possibly on the basis of more abstract value computations.

## Conflict of interest

The authors declare no conflict of interest.

## References

[bb0005] Barrash J., Tranel D., Anderson S.W. (2000). Acquired personality disturbances associated with bilateral damage to the ventromedial prefrontal region. Dev. Neuropsychol..

[bb0010] Basten U., Biele G., Heekeren H.R., Fiebach C.J. (2010). How the brain integrates costs and benefits during decision making. Proc. Natl. Acad. Sci. U. S. A..

[bb0015] Bechara A., Damasio A.R., Damasio H., Anderson S.W. (1994). Insensitivity to future consequences following damage to human prefrontal cortex. Cognition.

[bb0020] Bechara A., Tranel D., Damasio H. (2000). Characterization of the decision-making deficit of patients with ventromedial prefrontal cortex lesions. Brain.

[bb0025] Behrens T.E., Hunt L.T., Woolrich M.W., Rushworth M.F. (2008). Associative learning of social value. Nature.

[bb0035] Boorman E.D., Behrens T.E., Woolrich M.W., Rushworth M.F. (2009). How green is the grass on the other side? Frontopolar cortex and the evidence in favor of alternative courses of action. Neuron.

[bb0030] Boorman E.D., Behrens T.E., Rushworth M.F. (2011). Counterfactual choice and learning in a neural network centered on human lateral frontopolar cortex. PLoS Biol..

[bb0040] Cai X., Kim S., Lee D. (2011). Heterogeneous coding of temporally discounted values in the dorsal and ventral striatum during intertemporal choice. Neuron.

[bb0045] Damoiseaux J.S., Rombouts S.A., Barkhof F., Scheltens P., Stam C.J., Smith S.M., Beckmann C.F. (2006). Consistent resting-state networks across healthy subjects. Proc. Natl. Acad. Sci. U. S. A..

[bb0050] Deichmann R., Gottfried J.A., Hutton C., Turner R. (2003). Optimized EPI for fMRI studies of the orbitofrontal cortex. Neuroimage.

[bb0055] Dorris M.C., Glimcher P.W. (2004). Activity in posterior parietal cortex is correlated with the relative subjective desirability of action. Neuron.

[bb0060] Fearnley J.M., Lees A.J. (1991). Ageing and Parkinson's disease: substantia nigra regional selectivity. Brain.

[bb0065] Fellows L.K. (2006). Deciding how to decide: ventromedial frontal lobe damage affects information acquisition in multi-attribute decision making. Brain.

[bb0070] FitzGerald T.H., Seymour B., Dolan R.J. (2009). The role of human orbitofrontal cortex in value comparison for incommensurable objects. J. Neurosci..

[bb0075] Gold J.I., Shadlen M.N. (2007). The neural basis of decision making. Annu. Rev. Neurosci..

[bb0080] Hare T.A., Camerer C.F., Rangel A. (2009). Self-control in decision-making involves modulation of the vmPFC valuation system. Science.

[bb0085] Hare T.A., Malmaud J., Rangel A. (2011). Focusing attention on the health aspects of foods changes value signals in vmPFC and improves dietary choice. J. Neurosci..

[bb0090] Hare T.A., Schultz W., Camerer C.F., O'Doherty J.P., Rangel A. (2011). Transformation of stimulus value signals into motor commands during simple choice. Proc. Natl. Acad. Sci. U. S. A..

[bb0095] Hernandez A., Zainos A., Romo R. (2002). Temporal evolution of a decision-making process in medial premotor cortex. Neuron.

[bb0100] Hsu M., Krajbich I., Zhao C., Camerer C.F. (2009). Neural response to reward anticipation under risk is nonlinear in probabilities. J. Neurosci..

[bb0105] Hubbard E.M., Piazza M., Pinel P., Dehaene S. (2005). Interactions between number and space in parietal cortex. Nat. Rev. Neurosci..

[bb0110] Hunt L.T., Kolling N., Soltani A., Woolrich M.W., Rushworth M.F., Behrens T.E. (2012). Mechanisms underlying cortical activity during value-guided choice. Nat. Neurosci..

[bb0115] Jenkinson M. (2003). Fast, automated, N-dimensional phase-unwrapping algorithm. Magn. Reson. Med..

[bb0125] Jenkinson M., Smith S. (2001). A global optimisation method for robust affine registration of brain images. Med. Image Anal..

[bb0120] Jenkinson M., Bannister P., Brady M., Smith S. (2002). Improved optimization for the robust and accurate linear registration and motion correction of brain images. Neuroimage.

[bb0130] Jocham G., Hunt L.T., Near J., Behrens T.E. (2012). A mechanism for value-guided choice based on the excitation–inhibition balance in prefrontal cortex. Nat. Neurosci..

[bb0135] Kable J.W., Glimcher P.W. (2007). The neural correlates of subjective value during intertemporal choice. Nat. Neurosci..

[bb0140] Kim S., Hwang J., Lee D. (2008). Prefrontal coding of temporally discounted values during intertemporal choice. Neuron.

[bb0145] Kolling N., Behrens T.E., Mars R.B., Rushworth M.F. (2012). Neural mechanisms of foraging. Science.

[bb0150] Krajbich I., Armel C., Rangel A. (2010). Visual fixations and the computation and comparison of value in simple choice. Nat. Neurosci..

[bb0155] Lim S.L., O'Doherty J.P., Rangel A. (2011). The decision value computations in the vmPFC and striatum use a relative value code that is guided by visual attention. J. Neurosci..

[bb0160] Noonan M.P., Walton M.E., Behrens T.E., Sallet J., Buckley M.J., Rushworth M.F. (2010). Separate value comparison and learning mechanisms in macaque medial and lateral orbitofrontal cortex. Proc. Natl. Acad. Sci. U. S. A..

[bb0165] Öngür D., Price J.L. (2000). The organization of networks within the orbital and medial prefrontal cortex of rats, monkeys and humans. Cereb. Cortex.

[bb0170] Padoa-Schioppa C., Assad J.A. (2006). Neurons in the orbitofrontal cortex encode economic value. Nature.

[bb0175] Pessiglione M., Schmidt L., Draganski B., Kalisch R., Lau H., Dolan R.J., Frith C.D. (2007). How the brain translates money into force: a neuroimaging study of subliminal motivation. Science.

[bb0180] Pinel P., Piazza M., Le Bihan D., Dehaene S. (2004). Distributed and overlapping cerebral representations of number, size, and luminance during comparative judgments. Neuron.

[bb0185] Plassmann H., O'Doherty J., Rangel A. (2007). Orbitofrontal cortex encodes willingness to pay in everyday economic transactions. J. Neurosci..

[bb0190] Plassmann H., O'Doherty J.P., Rangel A. (2010). Appetitive and aversive goal values are encoded in the medial orbitofrontal cortex at the time of decision making. J. Neurosci..

[bb0195] Platt M.L., Glimcher P.W. (1999). Neural correlates of decision variables in parietal cortex. Nature.

[bb0200] Sack A.T. (2009). Parietal cortex and spatial cognition. Behav. Brain Res..

[bb0205] Serences J.T. (2008). Value-based modulations in human visual cortex. Neuron.

[bb0210] Shiner T., Seymour B., Wunderlich K., Hill C., Bhatia K.P., Dayan P., Dolan R.J. (2012). Dopamine and performance in a reinforcement learning task: evidence from Parkinson's disease. Brain.

[bb0215] Smith S.M., Jenkinson M., Woolrich M.W., Beckmann C.F., Behrens T.E., Johansen-Berg H., Bannister P.R., De Luca M., Drobnjak I., Flitney D.E. (2004). Advances in functional and structural MR image analysis and implementation as FSL. Neuroimage.

[bb0220] Sokol-Hessner P., Hutcherson C., Hare T., Rangel A. (2012). Decision value computation in DLPFC and VMPFC adjusts to the available decision time. Eur. J. Neurosci..

[bb0225] Sugrue L.P., Corrado G.S., Newsome W.T. (2004). Matching behavior and the representation of value in the parietal cortex. Science.

[bb0230] Tversky A., Kahneman D. (1992). Advances in prospect-theory—cumulative representation of uncertainty. J. Risk Uncertain..

[bb0235] Woolrich M.W., Ripley B.D., Brady M., Smith S.M. (2001). Temporal autocorrelation in univariate linear modeling of FMRI data. Neuroimage.

[bb0240] Wunderlich K., Rangel A., O'Doherty J.P. (2009). Neural computations underlying action-based decision making in the human brain. Proc. Natl. Acad. Sci. U. S. A..

[bb0245] Wunderlich K., Rangel A., O'Doherty J.P. (2010). Economic choices can be made using only stimulus values. Proc. Natl. Acad. Sci. U. S. A..

[bb0250] Yang T., Shadlen M.N. (2007). Probabilistic reasoning by neurons. Nature.

