# Supplementary material for: Dissociable contributions of ventromedial prefrontal and posterior parietal cortex to value-guided choice
Source: Neuroimage. 2014 Oct 15;100:498–506. doi: 10.1016/j.neuroimage.2014.06.005 (PMC4148525; doi:10.1016/j.neuroimage.2014.06.005)
Supplement: Supplementary file 1 — Supplementary material [file mmc1.pdf]

Supplementary information for

Dissociable contributions of ventromedial prefrontal and posterior parietal cortex to value-guided choice

Gerhard Jocham[1], P Michael Furlong[4], Inga L Kröger[5], Martin C Kahn[6], Laurence T Hunt[1,2], Tim EJ Behrens[1,3]

1) FMRIB Centre, University of Oxford, John Radcliffe Hospital, Oxford OX3 9DU, United Kingdom
2) Department of Experimental Psychology, University of Oxford, South Parks Road, Oxford OX1 3UD, United Kingdom
3) Wellcome Trust Centre for Neuroimaging, University College London, 12 Queen Square, London WC1N 3BG
4) The Robotics Institute, Carnegie Mellon University, Pittsburgh, PA, USA
5) Department of Systems Neuroscience, University of Hamburg, UKE, Martinistrasse 52, 20246 Hamburg, Germany
6) Graduate Programme in Neuroscience, University of Oxford

To whom correspondence should be addressed: gjocham@fmrib.ox.ac.uk

Supplementary results, supplementary references

Supplementary Figures 1 – 2

Supplementary Table 1

Supplementary results

In addition to our main regions of interest, we also tested for differences between conditions in MCC and PCC. The MCC followed similar temporal dependencies as pSPL. A value difference correlate was found in *short* and *middle* ($t_{27} > 2.09$, $p < 0.025$), but not in *long* ($p > 0.29$, supplementary Fig. S1), and this correlation tended to be more pronounced in *short* compared to *long* ($t_{27} > 1.55$, $p = 0.066$). Notably, further decomposition revealed that a significant negative unchosen value effect could be observed for both *short* and *middle* ($t_{27} > 2.03$, $p = 0.027$) but not long ($p > 0.44$). In contrast, a significant positive chosen value effect was only present as a trend for short ($t_{27} = 1.46$, $p = 0.078$) but not for middle and long ($p > 0.22$, supplementary Fig. S1). Thus, value difference coding in MCC was primarily driven by an unchosen value effect, with only a weak contribution of chosen value coding in *short*. In PCC, finally, robust value difference coding was present in all three conditions ($t_{27} > 1.95$, $p < 0.031$, supplementary Fig. S2) and this was more pronounced in *middle* compared to *long* ($t_{27} > 2.49$, $p = 0.019$, supplementary Fig. S2). A chosen value effect was present in all conditions ($t_{27} > 2.37$, $p = 0.015$). In contrast, a negative unchosen value effect was only present in middle ($t_{27} = 5.25$, $p < 0.00001$) and short ($t_{27} = 1.86$, $p = 0.038$), but not in long ($p=0.13$).

Thus, while the MCC followed the same pattern of temporal dependence as pSPL, PCC did not show such dependence at all. Instead, in PCC, a value difference signal was observed in all conditions, irrespective of the temporal requirements. The functional significance of these findings is unclear. PCC has been found to correlate with the subjective value of the chosen option in both fMRI (Kable and Glimcher, 2007; Levy and Glimcher, 2011) and single unit studies (McCoy and Platt, 2005); it is also typically found as part of the default mode network that also includes vmPFC (Gusnard et al., 2001; Raichle et al., 2001), but functional dissociations between these two interconnected regions have been relatively sparse.

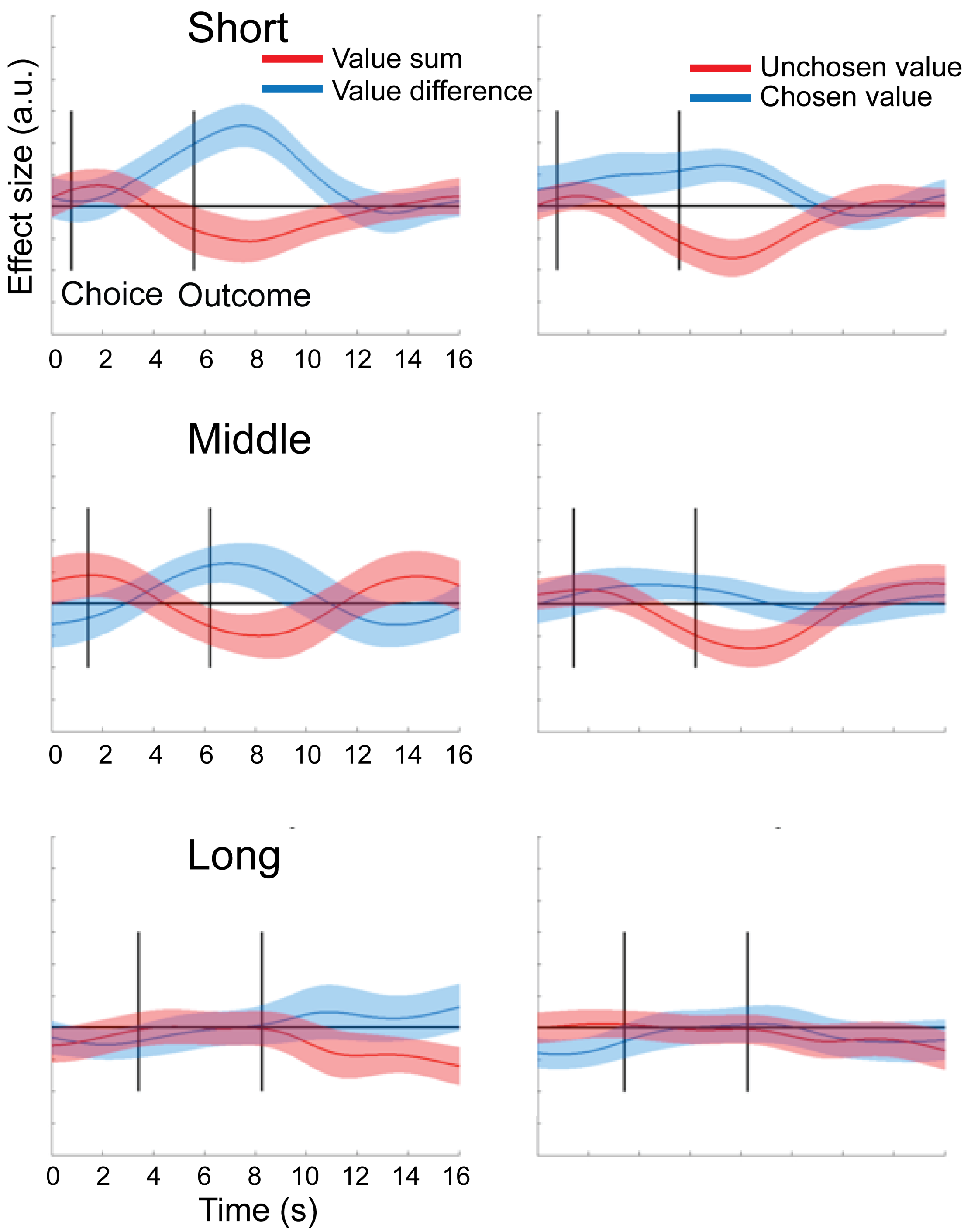

Figure S1) Timecourse of value-related effects in the midcingulate cortex (MCC) over the course of a trial in the three conditions. Left column: effects of value difference and value sum on BOLD activity in MCC. Right column: Effects of chosen and unchosen option value on MCC BOLD activity. Solid lines represent mean effect sizes across participants, shaded areas are standard error of the mean.

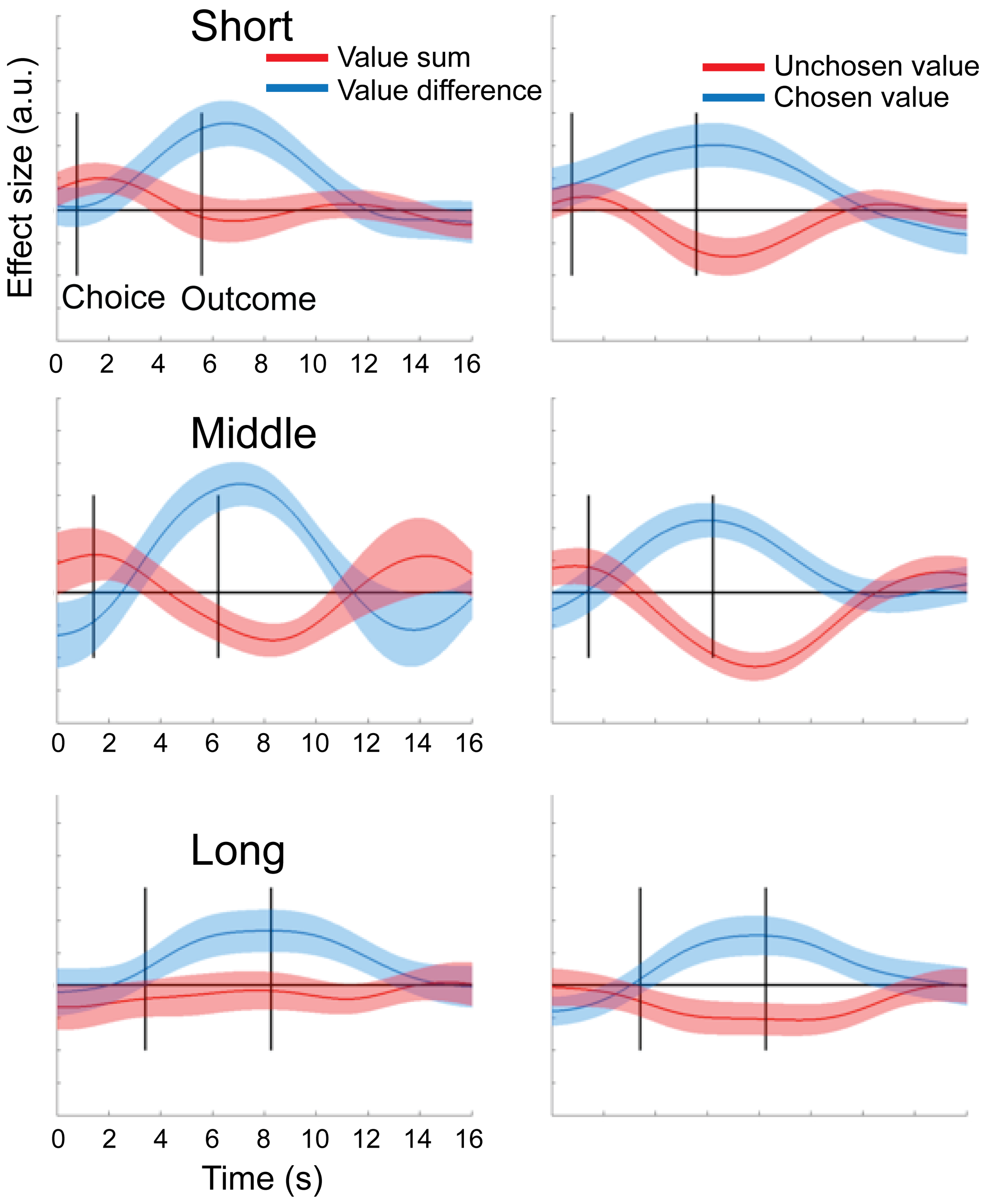

Figure S2) Timecourse of value-related effects in the posterior cingulate cortex (PCC) over the course of a trial in the three conditions. Left column: effects of value difference and value sum on BOLD activity in PCC. Right column: Effects of chosen and unchosen option value on PCC BOLD activity. Solid lines represent mean effect sizes across participants, shaded areas are standard error of the mean.

Table S1: All activations for the contrast "value difference" and "inverse value difference (= – negative correlation with value difference) obtained at $p < 0.001$, uncorrected.

Contrast	Anatomical region	Cluster size (mm3)	Maximum Z score	Peak coordinates (MNI xyz)
**Value Difference**	right TPJ	303	4.08	54, –38, 22
	left posterior superior temporal sulcus	264	4.18	–50, –54, 18
	left TPJ (Heschl Gyrus?)	148	3.9	–54, –32, 22
	right anterior occipital sulcus	130	4.16	54, –56, 10
	BA 25/32 / subgenual anterior cingulate cortex	91	4.66	4, 24, –6
	left insular cortex	75	4.1	–46, –2, –8
	right insular cortex	69	3.85	46, –2, 4
	pregenual anterior cingulate cortex	52	4.02	0, 38, 0
	ventromedial prefrontal cortex	52	4.03	–2, 28, –18
	posterior cingulate cortex	50	3.73	–6, –46, 34
	left rostral middle temporal gyrus	50	3.63	–60, –6, –16
	left parahippocampal gyrus	34	3.86	–32, –44, –8
	left anterior occipital sulcus	32	3.46	–48, –68, 12
	precuneus	31	3.5	–8, –58, 18
	right rostral superior temporal sulcus	27	3.65	50, –2, –16
	right parahippocampal gyrus	16	3.79	34, –44, –8

**Inverse Value Difference**	right intraparietal sulcus	5008	5.97	46, –36, 46
	left occipital cortex	3740	5.68	–30, –96, –10
	right anterior insula	2632	5.85	36, 20, –8
	medial prefrontal cortex (anterior midcingulate cortex)	2502	7.06	–4, 26, 38
	GPi / caudate (bilateral)	1090	4.66	–10, –2, 0
	left inferior frontal sulcus	874	5.19	–40, 2, 30
	left anterior insula	742	5.49	–32, 16, 2
	right superior frontal sulcus	721	5.4	30, 0, 52
	left cerebellum	275	4.51	–8, –74, –34
	lateral midbrain (right)	177	4.3	10, –28, –10
	right calcarine sulcus	171	3.64	14, –72, 6
	left inferior frontal gyrus	160	4.2	–44, 36, 14
	right cerebellum	150	4.51	8, –76, –28
	left BA 11	111	4	–40, 44, –10
	left calcarine sulcus	57	3.55	–10, –80, –2
	right lateral orbitofrontal cortex	48	4.23	26, 46, –16
	left lateral orbitofrontal cortex	20	3.57	–22, 44, –16

Supplementary references

Gusnard DA, Raichle ME, Raichle ME (2001) Searching for a baseline: functional imaging and the resting human brain. Nature reviews Neuroscience 2:685-694.

Kable JW, Glimcher PW (2007) The neural correlates of subjective value during intertemporal choice. Nat Neurosci 10:1625-1633.

Levy DJ, Glimcher PW (2011) Comparing apples and oranges: using reward-specific and reward-general subjective value representation in the brain. J Neurosci 31:14693-14707.

McCoy AN, Platt ML (2005) Risk-sensitive neurons in macaque posterior cingulate cortex. Nat Neurosci 8:1220-1227.

Raichle ME, MacLeod AM, Snyder AZ, Powers WJ, Gusnard DA, Shulman GL (2001) A default mode of brain function. Proc Natl Acad Sci U S A 98:676-682.
